# Piperacillin/Tazobactam Susceptibility Test Interpretive Criteria for Enterobacterales: Recommendations From the United States Committee on Antimicrobial Susceptibility Testing

**DOI:** 10.1093/cid/ciae328

**Published:** 2024-06-21

**Authors:** Thomas P Lodise, Sujata M Bhavnani, Paul G Ambrose, Helio S Sader, David Andes, Jason M Pogue

**Affiliations:** Department of Pharmacy Practice, Albany College of Pharmacy and Health Sciences, Albany, New York, USA; Institute for Clinical Pharmacodynamics, Inc, Schenectady, New York, USA; Institute for Clinical Pharmacodynamics, Inc, Schenectady, New York, USA; JMI Laboratories, North Liberty, Iowa, USA; Department of Medicine, Department of Medical Microbiology and Immunology, School of Medicine and Public Health and School of Pharmacy, University of Wisconsin–Madison, Madison, Wisconsin, USA; Department of Clinical Pharmacy, College of Pharmacy, University of Michigan, Ann Arbor, Michigan, USA

**Keywords:** susceptibility, piperacillin/tazobactam, Enterobacterales, ESBL, AmpC

## Abstract

The in vitro susceptibility testing interpretive criteria (STIC) for piperacillin/tazobactam (TZP) against Enterobacterales were recently updated by the US Food and Drug Administration, Clinical and Laboratory Standards Institute, and European Committee on Antimicrobial Susceptibility Testing. The United States Committee on Antimicrobial Susceptibility Testing (USCAST) also recently reviewed TZP STIC for Enterobacterales and arrived at different STIC for Enterobacterales. Here, we explain our recommendations and rationale behind them. Based on our review of the available data, USCAST does not recommend TZP STIC for certain Enterobacterales species that have a moderate to high likelihood of clinically significant AmpC production (*Enterobacter cloacae*, *Citrobacter freundii*, and *Klebsiella aerogenes* only) or for third-generation cephalosporin-nonsusceptible Enterobacterales. USCAST recommends a TZP susceptibility breakpoint of ≤ 16/4 mg/L for third-generation cephalosporin-susceptible Enterobacterales and only endorses the use of extended infusion TZP regimens for patients with infections due to these pathogens.

## Teaser

Contrary to other breakpoint (BP) setting organizations, USCAST does not recommend piperacillin/tazobactam (TZP) susceptibility BPs for Enterobacterales nonsusceptible to third-generation cephalosporins (3GC) or Enterobacterales with a high likelihood of significant AmpC production. USCAST only recommends TZP BPs for 3GC-susceptible Enterobacterales.

**(See the Editorial Commentary by Humphries on pages 1363–5.)**

Piperacillin/tazobactam (TZP) is recommended as a first-line treatment for Enterobacterales infections [1–3]. Despite its wide-scale use [4], there has been considerable debate on its role for infections caused by extended-spectrum β-lactamase (ESBL) and AmpC-producing Enterobacterales [5–7]. Data indicate that 15%–20% of *Escherichia coli* and *Klebsiella* spp. in the United States are third-generation cephalosporin-nonsusceptible (3GC-NS) [8–11], a phenotypic marker of ESBL expression, and that a majority of these isolates harbor CTX-M enzymes [12]. Although tazobactam is a potent inhibitor of most CTX-M enzymes [13–15], TZP has variable in vitro activity against ESBL-producing Enterobacterales [16–18]. The reduced TZP susceptibility against ESBL-producing Enterobacterales is multifactorial but driven in large part by the copresence of other β-lactamases [16, 18]. Concerns have also been raised regarding the use of TZP for Enterobacterales (eg, *Enterobacter cloacae*, *Citrobacter freundii*, and *Klebsiella aerogenes*) that have a moderate to high likelihood of clinically significant AmpC production, given that tazobactam does not efficiently inhibit these enzymes [5, 19].

The in vitro susceptibility testing interpretive criteria (STIC) for TZP against Enterobacterales were recently updated by the US Food and Drug Administration (FDA) [20], Clinical and Laboratory Standards Institute (CLSI) [21, 22], and European Committee on Antimicrobial Susceptibility Testing (EUCAST) [23] (Tables 1 and 2). The United States Committee on Antimicrobial Susceptibility Testing (USCAST) also recently convened to review TZP STIC for Enterobacterales. While USCAST appreciates the expertise of these organizations, USCAST arrived at different TZP STIC for Enterobacterales. Here, we explain our recommendations (Table 1), the rationale behind them, and the future research that is needed to further inform these STIC. Of note, USCAST did not discuss the optimal method for incorporating their proposed TZP STIC across clinical microbiologic laboratories in their deliberations. USCAST recognizes that many laboratories use obsolete breakpoints for a variety of reasons [24] and recommends that clinicians work with their microbiologic departments to ensure the most clinically appropriate STIC are used for interpreting TZP susceptibility results.

**Table 1.** United States Committee on Antimicrobial Susceptibility Testing, US Food and Drug Administration, Clinical and Laboratory Standards Institute, and European Committee on Antimicrobial Susceptibility Testing Susceptibility Test Interpretive Criteria for Piperacillin/Tazobactam Against Enterobacterales

	Current STIC (µg/mL) by Organization									
Enterobacterales	United States Committee on Antimicrobial Susceptibility Testing		Clinical and Laboratory Standards Institute [21]			US Food and Drug Administration [20]^a^			European Committee on Antimicrobial Susceptibility Testing [23]^b^	
	Susceptible	Resistant	Susceptible	Susceptible, dose dependent	Resistant	Susceptible	Intermediate	Resistant	Susceptible	Resistant
All Enterobacterales	No recommended STIC		≤ 8/4^c^	16/4^d^	≥ 32/4	≤ 8/4	16/4	≥ 32/4	≤ 8/4	> 16/4
Enterobacterales that have a moderate to high likelihood of clinically significant AmpC production	No recommended STIC									
Third-generation cephalosporin-nonsusceptible Enterobacterales	No recommended STIC									
Third-generation cephalosporin-susceptible Enterobacterales that do not have a moderate to high likelihood of clinically significant AmpC production^e^	16/4^f^	> 16/4								

Abbreviations: STIC, susceptibility test interpretive criteria.

^a^Clinical efficacy was shown for *Escherichia coli* and *Klebsiella pneumoniae.*

^b^Minimum inhibitory concentration of 16 mg/L is an area of technical uncertainty.

^c^Based on labeled dosing of 3.375 g or 4.5 g every 6 hours administered over 0.5 hours.

^d^Susceptible dose dependent. Based on a dose of 4.5 g every 6 hours over 3 hours or 4.5 g every 8 hours administered over 4 hours.

^e^Enterobacterales that have a moderate to high likelihood of clinically significant AmpC production due to an inducible chromosomal *ampC* gene include *Enterobacter cloacae*, *Citrobacter freundii*, and *Klebsiella aerogenes*.

^f^This recommendation is based on a piperacillin/tazobactam dose of 4.5 g infused over 3 hours every 6 hours or 4.5 g infused over 4 hours every 8 hours.

**Table 2.** Previous Piperacillin/Tazobactam Susceptibility Testing Interpretative Criteria for Enterobacterales by Organization

Organization	Susceptible	Intermediate	Resistant
US Food and Drug Administration [20]	≤16/4	32–64	≥128/4
Clinical and Laboratory Standards Institute [21]	≤16/4	32–64	≥128/4
European Committee on Antimicrobial Susceptibility Testing [23]	≤8/4	16 (area of technical uncertainty)	>16/4
United States Committee on Antimicrobial Susceptibility Testing	Never addressed		

##  

### Recommendation 1

USCAST does not recommend TZP STIC for Enterobacterales with a moderate to high likelihood of clinically significant AmpC production due to an inducible chromosomal *ampC* gene. This recommendation includes *E. cloacae*, *C. freundii*, and *K. aerogenes* only.

### Rationale

This USCAST recommendation, which aligns with the recommendations of the current Infectious Diseases Society of America (IDSA) guidance on the treatment of antimicrobial-resistant gram-negative infections [5], was largely based on the high potential for selection of derepressed AmpC mutants during TZP treatment of patients with infections due to these pathogens and the lack of in vitro activity of TZP against these derepressed mutants. Tazobactam does not efficiently inhibit most AmpC β-lactamases [19, 25–27]. While the degree of AmpC production varies by Enterobacterales that possess a chromosomal *ampC* gene, there is a high potential for selection of derepressed AmpC mutants when administering a labile weak inducer such as piperacillin for treatment of infections due to *E. cloacae*, *C. freundii*, and *K. aerogenes* [6, 19, 28–32]. Although TZP is considered a weak AmpC inducer [33], derepressed mutants of these species are usually TZP-resistant [19].

The USCAST recommendation was also informed by the negative signals observed in the pilot (n = 72), multicentered, randomized, open-label trial that compared TZP with meropenem for definitive treatment of bloodstream infections caused by AmpC β-lactamase–producing Enterobacterales (MERINO-2) [34]. Overall, no significant difference in the primary composite failure outcome (30-day mortality, clinical failure, microbiological failure, or microbiological relapse) was observed between treatment groups. However, 53% of patients in the trial were infected with Enterobacterales that are unlikely to develop clinically significant AmpC expression (ie, *Citrobacter braakii*, *Morganella morganii*, *Serratia marcescens*, *Providencia* spp., or *Serratia* spp.) [28]. Among the subgroup of 32 patients with infections due to Enterobacterales with a moderate to high likelihood of clinically significant AmpC production (ie, *Enterobacter* spp.), a nonsignificantly higher proportion of patients in the TZP arm met the primary composite failure outcome (28% vs 7%, respectively; *P* = .14). These findings were consistent with those from a recent observational study that demonstrated significantly higher treatment failure rates among patients who received piperacillin ± tazobactam relative to those who received a carbapenem or cefepime for definitive treatment of wild-type AmpC β-lactamase–producing Enterobacterales bloodstream infections or pneumonia [35]. In this study, >75% of patients in the piperacillin group were infected with an Enterobacterales with a moderate to high likelihood of clinically significant AmpC production.

USCAST acknowledges that the results of several real-world observational studies have not conclusively demonstrated that there is a significant increase in failure with TZP relative to carbapenems for patients with these infections [36, 37]. However, these studies were small and suffered from significant confounding by indication where more severely ill patients received a carbapenem. These studies also included Enterobacterales species with a low risk of clinically significant AmpC production (eg, *S. marcescens*) and/or those that lacked a chromosomal AmpC enzyme altogether (eg, *Citrobacter koseri*), limiting their applicability. Of note, the USCAST recommendation only includes *E. cloacae*, *C. freundii*, and *K. aerogenes* and does not apply to other Enterobacterales (eg, *Hafnia alvei*, *Citrobacter youngae*, *Yersinia enterocolitica*) with a moderate to high likelihood of clinically significant AmpC production due to the dearth of TZP data against these pathogens. For use of TZP in the treatment of patients with infections due to these other AmpC β-lactamase–producing Enterobacterales with a moderate to high likelihood of clinically significant AmpC production, USCAST identified this as an area that merits further research.

### Recommendation 2

USCAST does not recommend STIC for TZP against 3GC-NS Enterobacterales.

### Rationale

**Figure 1.**
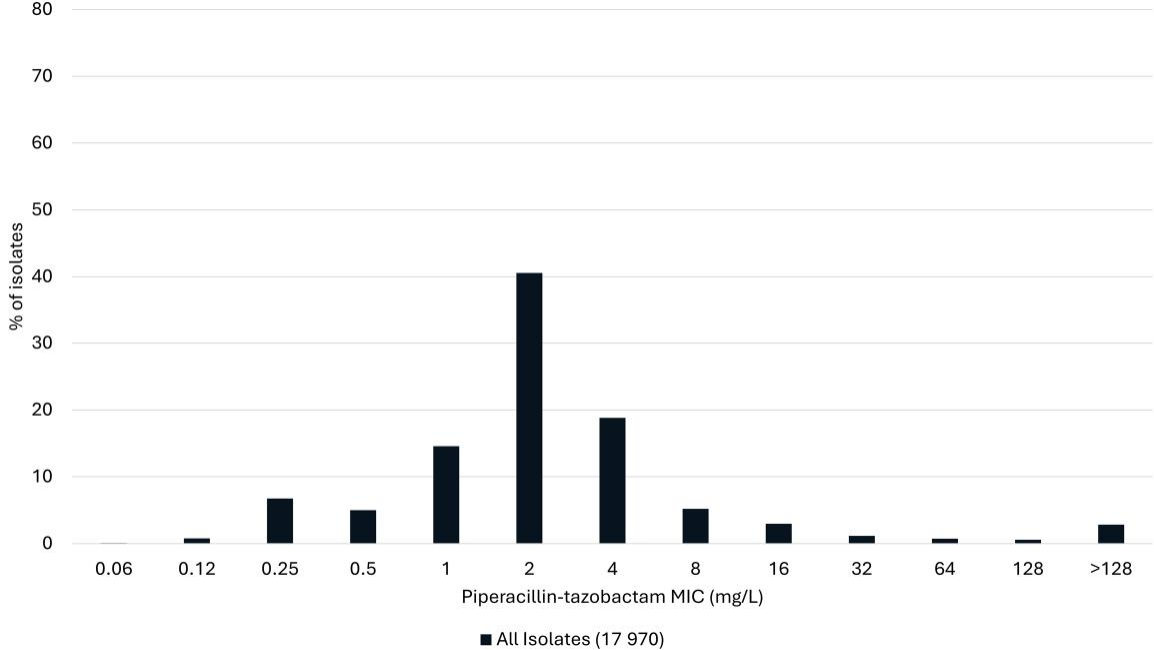
Piperacillin-tazobactam activity against Enterobacterales from US medical centers (2020–2022). Enterobacterales include *Escherichia coli* (n = 8750), *Klebsiella pneumoniae* (n = 5436), *Klebsiella oxytoca* (n = 1597), and *Proteus mirabilis* (n = 2187).

**Figure 2.**
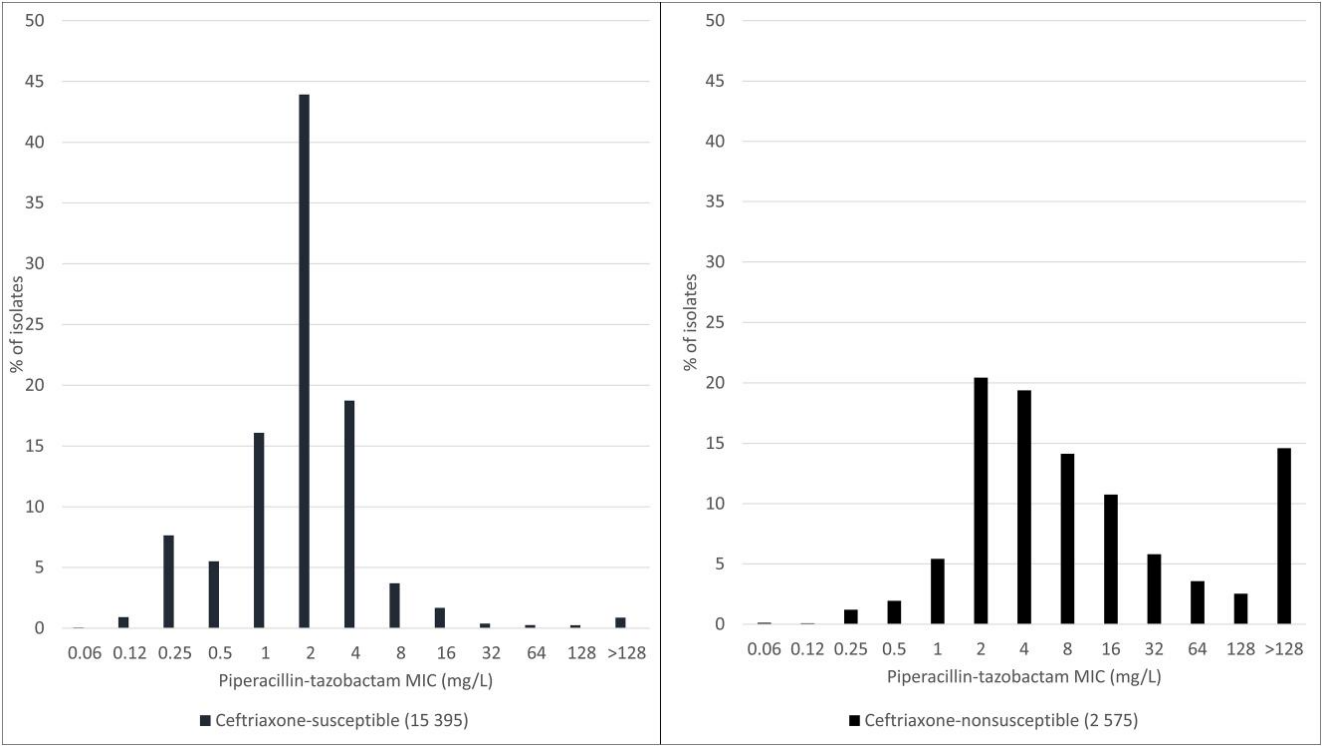
Piperacillin-tazobactam activity against Enterobacterales from US medical centers (2020–2022) stratified by ceftriaxone susceptibility. Enterobacterales include *Escherichia coli* (n = 8750), *Klebsiella pneumoniae* (n = 5436), *Klebsiella oxytoca* (n = 1597), and *Proteus mirabilis* (n = 2187). Enterobacterales were considered susceptible if the ceftriaxone MIC value was ≤1 mg/L. Enterobacterales were considered nonsusceptible if the ceftriaxone MIC value was ≥2 mg/L [21]. Abbreviation: MIC, minimum inhibitory concentration.

This USCAST recommendation was based on its review of available microbiological, preclinical, in silico, and clinical data (predominately bloodstream infection data) and is in concordance with the recent recommendations of the IDSA Gram-Negative Guidance Panel [38]. TZP minimum inhibitory concentration (MIC) data for Enterobacterales from the US SENTRY Antimicrobial Surveillance Program (2020–2022) are shown in Figure 1. The tentative epidemiological cutoff (ECOFF) values for thresholds from 95.0% to 99.9% were calculated for *Proteus mirabilis*, *E. coli*, *Klebsiella oxytoca*, and *Klebsiella pneumoniae* and ranged from 0.5 to 1, 4 to 8, 4 to 8, and 8 to 16 mg/L, respectively (Supplementary Table 1). While these data suggest the ECOFF can be used to help inform the TZP susceptibility breakpoint for Enterobacterales, there were stark differences in the TZP MIC distributions between ceftriaxone-nonsusceptible (CRO-NS) and ceftriaxone-susceptible (CRO-S) isolates (Figure 2, Supplementary Figures 1–3). USCAST believed the highly discordant TZP MIC distributions between CRO-NS vs CRO-S Enterobacterales isolates limited the utility of the ECOFF in informing the TZP susceptibility breakpoint against 3GC-NS Enterobacterales.

Available preclinical pharmacokinetic/pharmacodynamic (PK/PD) data suggest that currently approved TZP dosing schemes are inadequate against the range of MIC values currently considered susceptible for 3GC-NS Enterobacterales. Using a piperacillin 50% free time above the MIC (piperacillin 50% *f*T > MIC) as the PK/PD target associated with efficacy for TZP, most studies demonstrate that the probability of achieving this PK/PD target with TZP administered as a 0.5-hour intermittent infusion or ≥ 3-hour extended infusion is >90% for pathogens with MIC values ≤ 8/4 mg/L and ≤16/4 mg/L, respectively [39–44]. Although organizations have used this as part of their STIC justifications, there are 2 main issues with limiting the PK/PD assessment to this target. First, while generally accepted, data to support this *f*T > MIC target for piperacillin are lacking [20]. Second, in addition to the piperacillin probabilty of target attainment profile, tazobactam exposures are a critical determinant in defining the PK/PD profile of TZP against 3GC-NS Enterobacterales as piperacillin is readily hydrolyzed by ESBLs. Data from hollow fiber infection models of ESBL-producing Enterobacterales infections indicate tazobactam exposures associated with intermittent-infusion or extended-infusion TZP regimens are insufficient for restoring the activity of piperacillin against 3GC-NS *E. coli* and *Klebsiella* spp. within the range of TZP MIC values currently considered susceptible by the FDA, CLSI, and EUCAST [20, 21, 23, 45, 46].

The failure demonstrated in these models can be explained by a close assessment of tazobactam PK/PD. The most informative preclinical PK/PD assessment of tazobactam was a 1-compartment in vitro infection model that examined a range of tazobactam doses in combination with piperacillin 4 g IV (intravenous; 0.5 hour) every 6 hours against 3 ESBL-producing strains of Enterobacterales (1 *E. coli* and 2 *K. pneumoniae*) with TZP MIC values of 4/4 mg/L, 2/4 mg/L, and 1/4 mg/L [47]. In this analysis, the percentage of time during the dosing interval that free tazobactam concentrations exceeded the TZP MIC value was identified as the PK/PD index most associated with activity. Importantly, free tazobactam concentrations had to exceed the TZP MIC value for 64% and 77% of the dosing interval to achieve net bacterial stasis and 1 log_10_ colony-forming unit reduction, respectively, when tazobactam was administered with piperacillin 4 g IV (0.5 hour) every 6 hours. In Monte Carlo simulations of critically ill [48] patients with estimated creatinine clearances (CL_CR_) of 60–100 mL/min [27], the probabilities of achieving these critical tazobactam exposures associated with stasis and 1 log_10_ killing for TZP 4.5 g IV every 6 hours administered as an 0.5- or 3-hour infusion were less than approximately 90% for 3G-R Enterobacterales with TZP MIC values greater than 4/4 mg/L and 2/4 mg/L, respectively, depending on infusion duration and CL_CR_ (Supplementary Table 2). Considering that >50% of the observed TZP MIC values among 3G-R Enterobacterales in SENTRY were ≥4/4 mg/L (Figure 2), USCAST believes that even the most favorable interpretation of the in silico data [27] does not support the use of TZP in the treatment of patients with 3GC-NS Enterobacterales infections.

The USCAST recommendation was also informed by the MERINO trial [49], which compared definitive treatment with meropenem 1 g every 8 hours (0.5-hour infusion) or TZP 4.5 g every 6 hours (0.5-hour infusion) in adult patients with TZP-S, ceftriaxone-R (CRO-R) *E. coli*, or *K. pneumoniae* bloodstream infections. In this international, multicenter, open-label, randomized clinical trial, 30-day mortality was higher in the TZP arm relative to the meropenem arm (12.3% vs 3.7%, respectively; risk difference, 8.6%; 1-sided 97.5% confidence interval [CI], −α to 14.5%; *P* = .90 for noninferiority) [49]. Similar trends in favor of meropenem were demonstrated in the secondary outcomes of clinical cure, microbiological cure, and the development of resistance [49].

To investigate the potential reason(s) for the observed 30-day mortality differences between treatments in MERINO-1, post hoc analyses were performed, and an unexpectedly high rate of nonsusceptibility to TZP was demonstrated using broth microdilution at the central laboratory compared to Vitek or disk diffusion at the local site [50]. When the analysis was limited to patients with TZP-susceptible isolates via broth microdilution by the current FDA, CLSI, and EUCAST TZP susceptible breakpoint of ≤8/4 mg/L [20, 21, 23], 30-day mortality was still higher with TZP but no longer reached statistical significance (9% vs 5%, respectively; 95% CI, −2% to 11%). In the multivariate regression analyses, TZP MIC >16/4 mg/L was identified as the TZP MIC threshold best associated with 30-day mortality (30-day mortality was 50% [5 of 10] in patients with isolates that had TZP MIC values >16/4 mg/L vs 9% [13 of 147] in patients with isolates that had TZP MIC values ≤16/4 mg/L) [50].

While the findings from these post hoc clinical analyses support the FDA, CLSI, and EUCAST TZP susceptibility breakpoints [20, 21, 23], USCAST did not find them to be sufficient for endorsing a TZP susceptibility breakpoint of ≤8/4 mg/L for several reasons. First, initial isolates were only available in 84% of patients for the post hoc analysis, and 30-day mortality was higher among patients in the TZP arm with nonavailable vs available isolates (16.7% vs 11.5%, respectively) [50]. USCAST was concerned that this may have biased the findings in favor of TZP. Second, closer inspection of the 30-day mortality data by TZP MIC demonstrated that mortality exceeded 10% in TZP-treated patients with CRO-R Enterobacterales that had a TZP MIC value of 2 mg/L. Among, TZP-treated patients with TZP MIC values ≤1 mg/L, 30-day mortality exceeded 25%. This “u-shaped” mortality curve as a function of increasing TZP MIC values weakens any association between TZP MIC and outcome in this study [50]. Third, although not powered to examine subgroups, the differences in 30-day mortality between TZP- and meropenem-treated patients in the original analyses were considerably more pronounced in sicker and/or more complicated populations [49]. Despite the well-recognized limitations associated with subgroup analyses, USCAST believed these populations were more representative of patients encountered in practice with 3GC-NS Enterobacterales bloodstream infections [51].

The USCAST recommendation was not unanimous, and 1 dissenting voter was concerned the available data were predominately related to patients with bloodstream infections and did not rule out the potential role of TZP for the treatment of patients with complicated urinary tract infections (cUTIs) due to 3GC-NS Enterobacterales as there is some evidence that supports TZP usage in this setting [52–55]. However, there are significant limitations to the observational studies that have addressed the role of TZP for cUTIs, most notably confounding by indication [52, 54, 55], and the lone cUTI randomized clinical trial had a small sample size, limiting interpretation [53]. Additionally, the tazobactam PK/PD concerns previously described remain relevant to cUTIs. While USCAST acknowledges that the role of TZP for 3GC-NS Enterobacterales in cUTIs remains unresolved, USCAST believed the most prudent recommendation at this time was not to have a TZP susceptibility breakpoint for 3GC-NS Enterobacterales given the uncertainties regarding the effectiveness of TZP in these patients.

The USCAST members were in full agreement that additional preclinical PK/PD studies with a more diverse group of 3GC-NS Enterobacterales isolates are needed to better understand the PK/PD of TZP against 3GC-NS Enterobacterales. If TZP use is supported by additional preclinical evidence, further randomized clinical trials would then be warranted to better quantify the efficacy of TZP for patients with 3GC-NS Enterobacterales infections. As part of these proposed studies, TZP should be evaluated in patients with less invasive 3GC-NS Enterobacterales infections, such as cUTIs, given the commonality of these infections and uncertainty of TZP's role for these patients. Of note, PETERPEN [56] is an ongoing, open-label, randomized clinical trial comparing extended-infusion TZP and meropenem for ESBL Enterobacterales bloodstream infections, and results of this study will help inform this conversation.

### Recommendation 3

USCAST recommends that the STIC for TZP against 3GC-S Enterobacterales that do not have a moderate to high likelihood of clinically significant AmpC production due to an inducible chromosomal *AmpC* gene are susceptible at MIC values ≤16/4 mg/L and resistant at MIC values >16/4 mg/L. This recommendation is based on TZP dosing regimens administered as an extended infusion (4.5 g infused over 3 hours every 6 hours or 4.5 g infused over 4 hours every 8 hours).

### Rationale

The USCAST recommendations were primarily based on review of available microbiological and in silico data. TZP MIC distribution data from the SENTRY Antimicrobial Surveillance Program for CRO-S Enterobacterales (Figure 2) indicate that more than 95% of isolates have a TZP MIC ≤ 8/4 mg/L, supporting the FDA, CLSI, and EUCAST susceptibility breakpoint of ≤ 8/4 mg/L [20, 21, 23]. However, nearly 5% of CRO-S *K. pneumoniae* had a TZP MIC of 16/4 mg/L (Supplementary Figure 2), and USCAST therefore deemed that a 3GC-S Enterobacterales susceptibility breakpoint of ≤16/4 mg/L would be preferred if pharmacokinetically justified. USCAST endorses 2 extended-infusion TZP regimens (ie, 4.5 g infused over 3 hours every 6 hours or 4.5 g infused over 4 hours every 8 hours) for its proposed 3GC-S Enterobacterales susceptibility breakpoint given that the results of published target attainment analyses indicate the probability of achieving 50% piperacillin *f*T > MIC with these extended-infusion TZP regimens is >90% for pathogens with TZP MIC values ≤ 16 mg/L [40, 42, 57, 58]. Of note, these are the same 2 extended-infusion TZP regimens recommended by CLSI for Enterobacterales with MIC values of 16/4 mg/L (susceptible-dose dependent) [21, 22]. However, UCSAST preferentially recommends TZP 4.5 g (3-hour infusion) every 6 hours for patients with CL_CR_ ≥ 100 mL/min based on Monte Carlo simulation studies that evaluated the effect of varying CL_CR_ on the observed probabilities of achieving 50% piperacillin *f*T > MIC (Supplementary Table 3) [57, 59]. USCAST was opposed to the use of intermittent infusion TZP (4.5 g IV over 0.5 hours every 6 hours) for patients with 3GC-S Enterobacterales infections as the probability of achieving 50% *f*T > MIC was <90% in simulated patients with (1) CL_CR_ ≥60 mL/min and TZP MIC of ≥8/4 mg/L, (2) CL_CR_ ≥80 mL/min and TZP MIC of ≥4/4 mg/L, and (3) CL_CR_ ≥100 mL/min and TZP MIC of ≥2/4 mg/L (Supplementary Table 3) [57, 59].

**Figure 3.**
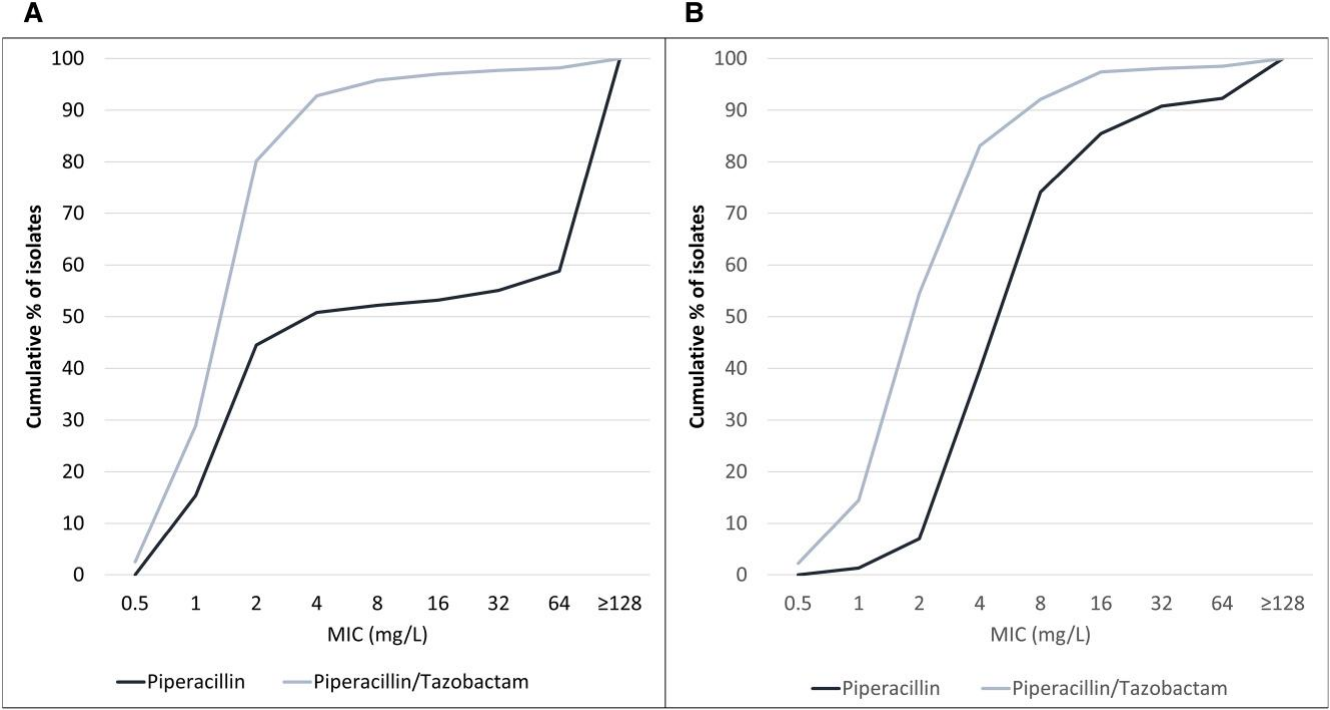
Activity of piperacillin and piperacillin/tazobactam against ceftriaxone-susceptible *Escherichia coli* (*A*) (4867 isolates) and ceftriaxone-susceptible *Klebsiella pneumoniae* (*B*) (2783 isolates) from North America medical centers (2007–2010). Enterobacterales were considered susceptible if the ceftriaxone MIC value was ≤1 mg/L. Abbreviation: MIC, minimum inhibitory concentration.

While piperacillin retains activity against most 3GC-S Enterobacterales, data from the SENTRY Antimicrobial Surveillance Program (2007– 2010) demonstrate that the addition of tazobactam increases the piperacillin susceptibility rates (at a breakpoint of ≤16/4 mg/L) from 86% to 97% for *K. pneumoniae* and from 53% to 97% for *E. coli* (Figure 3). Thus, the presence of tazobactam is not immaterial to the considerations that surround TZP breakpoints for 3GC-S Enterobacterales. USCAST acknowledges that there are limited preclinical data that characterize the PK/PD targets associated with efficacy for piperacillin alone and piperacillin in the presence of tazobactam against 3GC-S Enterobacterales [20]. However, there are clinical data that suggest that critically ill patients with gram-negative infections who achieve 50% *f*T > MIC with piperacillin and other β-lactams are more likely to have a positive clinical outcome [60–62].

Despite the notable data gaps, USCAST was in favor of these breakpoint recommendations based on the belief that use of TZP will largely be empiric for patients with 3GC-S Enterobacterales infections and that good stewardship practices will foster deescalation in most circumstances to a narrower agent when 3GCs demonstrate susceptibility. It is important to note that the 3GC-S Enterobacterales susceptibility breakpoint of ≤16/4 mg/L is contingent upon use of extended-infusion TZP. If institutions find administration of extended-infusion TZP infeasible, a reasonable susceptibility breakpoint with intermittent-infusion TZP would be 8/4 mg/L, as recommended by the FDA, CLSI, and EUCAST [20, 21, 23]. However, the probability of achieving 50% piperacillin *f*T > MIC would be <90% for 3GC-S Enterobacterales with TZP MIC values ≤8 mg/L among some renal function subgroups with intermittent-infusion TZP (Supplementary Table 3) [57, 59]. Based on the Monte Carlo simulation studies that evaluated the effect of varying CL_CR_ on achieving 50% piperacillin *f*T > MIC [57, 59], USCAST was not in favor of endorsing a TZP STIC for 3GC-S Enterobacterales that included a susceptible breakpoint for intermittent-infusion TZP dosing. USCAST unanimously agreed that additional preclinical PK/PD studies are needed to assess the piperacillin and tazobactam PK/PD targets associated with efficacy and using such targets to determine TZP dosing schemes necessary to ensure piperacillin's activity against 3GC-S Enterobacterales.

## Supplementary Data

Supplementary materials are available at *Clinical Infectious Diseases* online. Consisting of data provided by the authors to benefit the reader, the posted materials are not copyedited and are the sole responsibility of the authors, so questions or comments should be addressed to the corresponding author.

## Supplementary Material

ciae328_Supplementary_Data

## Notes

***Disclaimer.*** This manuscript was submitted on behalf of the behalf of the United States Committee on Antimicrobial Susceptibility Testing.

## References

[1] Kalil AC, Metersky ML, Klompas M, et al Management of adults with hospital-acquired and ventilator-associated pneumonia: 2016 clinical practice guidelines by the Infectious Diseases Society of America and the American Thoracic Society. Clin Infect Dis 2016; 63:e61–111. 27418577 10.1093/cid/ciw353 PMC4981759

[2] Mazuski JE, Tessier JM, May AK, et al The Surgical Infection Society revised guidelines on the management of intra-abdominal infection. Surg Infect (Larchmt) 2017; 18:1–76. 28085573 10.1089/sur.2016.261

[3] Stevens DL, Bisno AL, Chambers HF, et al Practice guidelines for the diagnosis and management of skin and soft tissue infections: 2014 update by the Infectious Diseases Society of America. Clin Infect Dis 2014; 59:e10–52. 24973422 10.1093/cid/ciu444

[4] Goodman KE, Baghdadi JD, Magder LS, et al Patterns, predictors, and inter-center variability in empiric gram-negative antibiotic use across 928 U.S. hospitals. Clin Infect Dis 2022; 76:e1224–35. 10.1093/cid/ciac504 PMC9907550 35737945

[5] Tamma PD, Aitken SL, Bonomo RA, Mathers AJ, van Duin D, Clancy CJ. Infectious Diseases Society of America guidance on the treatment of AmpC β-lactamase-producing Enterobacterales, carbapenem-resistant *Acinetobacter baumannii*, and *Stenotrophomonas maltophilia* infections. Clin Infect Dis 2022; 74:2089–114. 34864936 10.1093/cid/ciab1013

[6] Tamma PD, Girdwood SC, Gopaul R, et al The use of cefepime for treating AmpC beta-lactamase-producing Enterobacteriaceae. Clin Infect Dis 2013; 57:781–8. 23759352 10.1093/cid/cit395

[7] Paul M, Carrara E, Retamar P, et al European Society of Clinical Microbiology and Infectious Diseases (ESCMID) guidelines for the treatment of infections caused by multidrug-resistant gram-negative bacilli (endorsed by European Society of Intensive Care Medicine). Clin Microbiol Infect 2022; 28:521–47. 34923128 10.1016/j.cmi.2021.11.025

[8] Weiner-Lastinger LM, Abner S, Edwards JR, et al Antimicrobial-resistant pathogens associated with adult healthcare-associated infections: summary of data reported to the National Healthcare Safety Network, 2015–2017. Infect Control Hosp Epidemiol 2020; 41:1–18. 31767041 10.1017/ice.2019.296 PMC8276252

[9] Weiner LM, Webb AK, Limbago B, et al Antimicrobial-resistant pathogens associated with healthcare-associated infections: summary of data reported to the National Healthcare Safety Network at the Centers for Disease Control and Prevention, 2011–2014. Infect Control Hosp Epidemiol 2016; 37:1288–301. 27573805 10.1017/ice.2016.174 PMC6857725

[10] Karlowsky JA, Lob SH, DeRyke CA, et al Prevalence of ESBL non-CRE *Escherichia coli* and *Klebsiella pneumoniae* among clinical isolates collected by the SMART Global Surveillance Programme from 2015 to 2019. Int J Antimicrob Agents 2022; 59:106535. 35091052 10.1016/j.ijantimicag.2022.106535

[11] Jernigan JA, Hatfield KM, Wolford H, et al Multidrug-resistant bacterial infections in U.S. hospitalized patients, 2012–2017. N Engl J Med 2020; 382:1309–19. 32242356 10.1056/NEJMoa1914433 PMC10961699

[12] Rossolini GM, D'Andrea MM, Mugnaioli C. The spread of CTX-M-type extended-spectrum beta-lactamases. Clin Microbiol Infect 2008; 14(Suppl 1):33–41. 18154526 10.1111/j.1469-0691.2007.01867.x

[13] Faheem M, Rehman MT, Danishuddin M, Khan AU. Biochemical characterization of CTX-M-15 from *Enterobacter cloacae* and designing a novel non-beta-lactam-beta-lactamase inhibitor. PLoS One 2013; 8:e56926. 23437273 10.1371/journal.pone.0056926 PMC3578935

[14] Walther-Rasmussen J, Høiby N. Cefotaximases (CTX-M-ases), an expanding family of extended-spectrum beta-lactamases. Can J Microbiol 2004; 50:137–65. 15105882 10.1139/w03-111

[15] Bush K, Macalintal C, Rasmussen BA, Lee VJ, Yang Y. Kinetic interactions of tazobactam with beta-lactamases from all major structural classes. Antimicrob Agents Chemother 1993; 37:851–8. 8388201 10.1128/aac.37.4.851 PMC187782

[16] Livermore DM, Day M, Cleary P, et al OXA-1 beta-lactamase and non-susceptibility to penicillin/beta-lactamase inhibitor combinations among ESBL-producing *Escherichia coli*. J Antimicrob Chemother 2019; 74:326–33. 30388219 10.1093/jac/dky453

[17] Kumar D, Singh AK, Ali MR, Chander Y. Antimicrobial susceptibility profile of extended spectrum beta-lactamase (ESBL) producing *Escherichia coli* from various clinical samples. Infect Dis (Auckl) 2014; 7:1–8. 24847178 10.4137/IDRT.S13820 PMC4024053

[18] Henderson A, Humphries R. Building a better test for piperacillin-tazobactam susceptibility testing: would that it were so simple (it's complicated). J Clin Microbiol 2020; 58:e01649-19. 10.1128/JCM.01649-19 PMC6989057 31723014

[19] Tamma PD, Doi Y, Bonomo RA, Johnson JK, Simner PJ. Antibacterial resistance leadership G. A primer on AmpC beta-lactamases: necessary knowledge for an increasingly multidrug-resistant world. Clin Infect Dis 2019; 69:1446–55. 30838380 10.1093/cid/ciz173 PMC6763639

[20] FDA Rationale for Piperacillin-Tazobactam Breakpoints for Enterobacterales. Available at: https://www.fda.gov/drugs/development-resources/fda-rationale-piperacillin-tazobactam-breakpoints-enterobacterales. Accessed 12 September 2023.

[21] Clinical and Laboratory Standards Institute. Performance standards for antimicrobial susceptibility testing. 33rd ed. CLSI Supplement M100. Wayne: Clinical and Laboratory Standards Institute, 2023.

[22] Tamma PD, Harris PNA, Mathers AJ, Wenzler E, Humphries RM. Breaking down the breakpoints: rationale for the 2022 Clinical and Laboratory Standards Institute revised piperacillin-tazobactam breakpoints against Enterobacterales. Clin Infect Dis 2022; 77:1585–90. 10.1093/cid/ciac688 36001445

[23] European Committee on Antimicrobial Susceptibility Testing . Breakpoint tables for interpretation of MICs and zone diameters. Version 12.0, 2022. Available at: http://www.eucast.org. Accessed 12 September 2023.

[24] Simner PJ, Rauch CA, Martin IW, et al Raising the bar: improving antimicrobial resistance detection by clinical laboratories by ensuring use of current breakpoints. Open Forum Infect Dis 2022; 9:ofac007. 35146049 10.1093/ofid/ofac007 PMC8826219

[25] Bush K, Bradford PA. Interplay between beta-lactamases and new beta-lactamase inhibitors. Nat Rev Microbiol 2019; 17:295–306. 30837684 10.1038/s41579-019-0159-8

[26] Drawz SM, Bonomo RA. Three decades of beta-lactamase inhibitors. Clin Microbiol Rev 2010; 23:160–201. 20065329 10.1128/CMR.00037-09 PMC2806661

[27] Monogue ML, Heil EL, Aitken SL, Pogue JM. The role of tazobactam-based combinations for the management of infections due to extended-spectrum beta-lactamase-producing Enterobacterales: insights from the Society of Infectious Diseases Pharmacists. Pharmacotherapy 2021; 41:864–80. 34689349 10.1002/phar.2623

[28] Kohlmann R, Bahr T, Gatermann SG. Species-specific mutation rates for ampC derepression in Enterobacterales with chromosomally encoded inducible AmpC beta-lactamase. J Antimicrob Chemother 2018; 73:1530–6. 29566147 10.1093/jac/dky084

[29] Choi SH, Lee JE, Park SJ, et al Emergence of antibiotic resistance during therapy for infections caused by Enterobacteriaceae producing AmpC beta-lactamase: implications for antibiotic use. Antimicrob Agents Chemother 2008; 52:995–1000. 18086837 10.1128/AAC.01083-07 PMC2258504

[30] Kaye KS, Cosgrove S, Harris A, Eliopoulos GM, Carmeli Y. Risk factors for emergence of resistance to broad-spectrum cephalosporins among Enterobacter spp. Antimicrob Agents Chemother 2001; 45:2628–30. 11502540 10.1128/AAC.45.9.2628-2630.2001 PMC90703

[31] Jacobson KL, Cohen SH, Inciardi JF, et al The relationship between antecedent antibiotic use and resistance to extended-spectrum cephalosporins in group I beta-lactamase-producing organisms. Clin Infect Dis 1995; 21:1107–13. 8589129 10.1093/clinids/21.5.1107

[32] Chow JW, Fine MJ, Shlaes DM, et al Enterobacter bacteremia: clinical features and emergence of antibiotic resistance during therapy. Ann Intern Med 1991; 115:585–90. 1892329 10.7326/0003-4819-115-8-585

[33] Weber DA, Sanders CC. Diverse potential of beta-lactamase inhibitors to induce class I enzymes. Antimicrob Agents Chemother 1990; 34:156–8. 2327752 10.1128/aac.34.1.156 PMC171539

[34] Stewart AG, Paterson DL, Young B, et al Meropenem versus piperacillin-tazobactam for definitive treatment of bloodstream infections caused by AmpC beta-lactamase-producing Enterobacter spp., *Citrobacter freundii*, *Morganella morganii*, *Providencia* spp, or *Serratia marcescens*: a pilot multicenter randomized controlled trial (MERINO-2). Open Forum Infect Dis 2021; 8:ofab387. 34395716 10.1093/ofid/ofab387 PMC8361238

[35] Maillard A, Delory T, Bernier J, et al Effectiveness of third generation cephalosporins or piperacillin compared to cefepime or carbapenems for severe infections caused by wild-type AmpC beta-lactamase-producing Enterobacterales: a multicenter retrospective propensity-weighted study. Int J Antimicrob Agents 2023; 62:106809. 37028731 10.1016/j.ijantimicag.2023.106809

[36] Herrmann L, Kimmig A, Rodel J, et al Early treatment outcomes for bloodstream infections caused by potential AmpC beta-lactamase-producing Enterobacterales with focus on piperacillin/tazobactam: a retrospective cohort study. Antibiotics (Basel) 2021; 10:665. 34199546 10.3390/antibiotics10060665 PMC8229083

[37] Cheng MP, Lee RS, Cheng AP, et al Beta-lactam/beta-lactamase inhibitor therapy for potential AmpC-producing organisms: a systematic review and meta-analysis. Open Forum Infect Dis 2019; 6:ofz248. 31363762 10.1093/ofid/ofz248 PMC6656656

[38] Tamma PD, Aitken SL, Bonomo RA, Mathers AJ, van Duin D, Clancy CJ. Infectious Diseases Society of America guidance on the treatment of extended-spectrum beta-lactamase producing Enterobacterales (ESBL-E), carbapenem-resistant Enterobacterales (CRE), and *Pseudomonas aeruginosa* with difficult-to-treat resistance (DTR-*P. aeruginosa*). Clin Infect Dis 2021; 72:e169–83. 33106864 10.1093/cid/ciaa1478

[39] El-Haffaf I, Caissy JA, Marsot A. Piperacillin-tazobactam in intensive care units: a review of population pharmacokinetic analyses. Clin Pharmacokinet 2021; 60:855–75. 33876381 10.1007/s40262-021-01013-1

[40] Thabit AK, Grupper M, Nicolau DP, Kuti JL. Simplifying piperacillin/tazobactam dosing: pharmacodynamics of utilizing only 4.5 or 3.375 g doses for patients with normal and impaired renal function. J Pharm Pract 2017; 30:593–9. 29121839 10.1177/0897190016684453

[41] Felton TW, Hope WW, Lomaestro BM, et al Population pharmacokinetics of extended-infusion piperacillin-tazobactam in hospitalized patients with nosocomial infections. Antimicrob Agents Chemother 2012; 56:4087–94. 22585219 10.1128/AAC.00521-12 PMC3421565

[42] Lodise TP, Lomaestro BM, Drusano GL, Society of Infectious Diseases Pharmacists. Application of antimicrobial pharmacodynamic concepts into clinical practice: focus on beta-lactam antibiotics: insights from the Society of Infectious Diseases Pharmacists. Pharmacotherapy 2006; 26:1320–32. 16945055 10.1592/phco.26.9.1320

[43] Li C, Kuti JL, Nightingale CH, Mansfield DL, Dana A, Nicolau DP. Population pharmacokinetics and pharmacodynamics of piperacillin/tazobactam in patients with complicated intra-abdominal infection. J Antimicrob Chemother 2005; 56:388–95. 16002420 10.1093/jac/dki243

[44] Lodise TP Jr, Lomaestro B, Rodvold KA, Danziger LH, Drusano GL. Pharmacodynamic profiling of piperacillin in the presence of tazobactam in patients through the use of population pharmacokinetic models and Monte Carlo simulation. Antimicrob Agents Chemother 2004; 48:4718–24. 15561849 10.1128/AAC.48.12.4718-4724.2004 PMC529233

[45] Abodakpi H, Chang KT, Gao S, Sanchez-Diaz AM, Canton R, Tam VH. Optimal piperacillin-tazobactam dosing strategies against extended-spectrum-beta-lactamase-producing Enterobacteriaceae. Antimicrob Agents Chemother 2019; 63:e01906-18. 30530606 10.1128/AAC.01906-18 PMC6355564

[46] Sumi CD, Heffernan AJ, Naicker S, et al Pharmacodynamic evaluation of intermittent versus extended and continuous infusions of piperacillin/tazobactam in a hollow-fibre infection model against *Escherichia coli* clinical isolates. J Antimicrob Chemother 2022; 77:3026–34. 36031790 10.1093/jac/dkac273

[47] VanScoy BDRC, McCauley J, et al Determination of the tazobactam exposure required for piperacillin efficacy using a one-compartment in vitro infection model. ASM Microbe 2016. Poster presentation (poster number 506) at American Society for Microbiology Microbe 2016, Boston, MA. 16–20 June 2016.

[48] Kalaria SN, Gopalakrishnan M, Heil EL. A population pharmacokinetics and pharmacodynamic approach to optimize tazobactam activity in critically ill patients. Antimicrob Agents Chemother 2020; 64:e02093-19. 31871076 10.1128/AAC.02093-19 PMC7038264

[49] Harris PNA, Tambyah PA, Lye DC, et al Effect of piperacillin-tazobactam vs meropenem on 30-day mortality for patients with *E coli* or *Klebsiella pneumoniae* bloodstream infection and ceftriaxone resistance: a randomized clinical trial. JAMA 2018; 320:984–94. 30208454 10.1001/jama.2018.12163 PMC6143100

[50] Henderson A, Paterson DL, Chatfield MD, et al Association between minimum inhibitory concentration, beta-lactamase genes and mortality for patients treated with piperacillin/tazobactam or meropenem from the MERINO study. Clin Infect Dis 2021; 73:e3842–50. 33106863 10.1093/cid/ciaa1479

[51] Gutierrez-Gutierrez B, Perez-Galera S, Salamanca E, et al A multinational, preregistered cohort study of beta-lactam/beta-lactamase inhibitor combinations for treatment of bloodstream infections due to extended-spectrum-beta-lactamase-producing Enterobacteriaceae. Antimicrob Agents Chemother 2016; 60:4159–69. 27139473 10.1128/AAC.00365-16 PMC4914653

[52] Sharara SL, Amoah J, Pana ZD, Simner PJ, Cosgrove SE, Tamma PD. Is piperacillin-tazobactam effective for the treatment of pyelonephritis caused by extended-spectrum beta-lactamase-producing organisms? Clin Infect Dis 2020; 71:e331–7. 31859352 10.1093/cid/ciz1205 PMC7643734

[53] Seo YB, Lee J, Kim YK, et al Randomized controlled trial of piperacillin-tazobactam, cefepime and ertapenem for the treatment of urinary tract infection caused by extended-spectrum beta-lactamase-producing *Escherichia coli*. BMC Infect Dis 2017; 17:404. 28592240 10.1186/s12879-017-2502-x PMC5463388

[54] Yoon YK, Kim JH, Sohn JW, Yang KS, Kim MJ. Role of piperacillin/tazobactam as a carbapenem-sparing antibiotic for treatment of acute pyelonephritis due to extended-spectrum beta-lactamase-producing *Escherichia coli*. Int J Antimicrob Agents 2017; 49:410–5. 28263710 10.1016/j.ijantimicag.2016.12.017

[55] Dizbay M, Ozger HS, Karasahin O, Karasahin EF. Treatment efficacy and superinfection rates in complicated urinary tract infections treated with ertapenem or piperacillin tazobactam. Turk J Med Sci 2016; 46:1760–4. 28081324 10.3906/sag-1506-157

[56] Bitterman R, Koppel F, Mussini C, et al Piperacillin-tazobactam versus meropenem for treatment of bloodstream infections caused by third-generation cephalosporin-resistant Enterobacteriaceae: a study protocol for a non-inferiority open-label randomised controlled trial (PeterPen). BMJ Open 2021; 11:e040210. 10.1136/bmjopen-2020-040210 PMC7871690 33558347

[57] Alobaid AS, Wallis SC, Jarrett P, et al Population pharmacokinetics of piperacillin in nonobese, obese, and morbidly obese critically ill patients. Antimicrob Agents Chemother 2017; 61:e01276-16. 28052849 10.1128/AAC.01276-16 PMC5328553

[58] Kim A, Sutherland CA, Kuti JL, Nicolau DP. Optimal dosing of piperacillin-tazobactam for the treatment of *Pseudomonas aeruginosa* infections: prolonged or continuous infusion? Pharmacotherapy 2007; 27:1490–7. 17963458 10.1592/phco.27.11.1490

[59] Patel N, Scheetz MH, Drusano GL, Lodise TP. Identification of optimal renal dosage adjustments for traditional and extended-infusion piperacillin-tazobactam dosing regimens in hospitalized patients. Antimicrob Agents Chemother 2010; 54:460–5. 19858253 10.1128/AAC.00296-09 PMC2798531

[60] Abdul-Aziz MH, Sulaiman H, Mat-Nor MB, et al Beta-lactam infusion in severe sepsis (BLISS): a prospective, two-centre, open-labelled randomised controlled trial of continuous versus intermittent beta-lactam infusion in critically ill patients with severe sepsis. Intensive Care Med 2016; 42:1535–45. 26754759 10.1007/s00134-015-4188-0

[61] Abdul-Aziz MH, Lipman J, Akova M, et al Is prolonged infusion of piperacillin/tazobactam and meropenem in critically ill patients associated with improved pharmacokinetic/pharmacodynamic and patient outcomes? An observation from the Defining Antibiotic Levels in Intensive care unit patients (DALI) cohort. J Antimicrob Chemother 2016; 71:196–207. 26433783 10.1093/jac/dkv288

[62] Roberts JA, Paul SK, Akova M, et al DALI: defining antibiotic levels in intensive care unit patients: are current beta-lactam antibiotic doses sufficient for critically ill patients? Clin Infect Dis 2014; 58:1072–83. 24429437 10.1093/cid/ciu027

