# Supplementary material for: Piperacillin/Tazobactam Susceptibility Test Interpretive Criteria for Enterobacterales: Recommendations From the United States Committee on Antimicrobial Susceptibility Testing
Source: Clin Infect Dis. 2024 Jun 21;79(6):1354–62. doi: 10.1093/cid/ciae328 (PMC11650869; doi:10.1093/cid/ciae328)
Supplement: ciae328_Supplementary_Data [file ciae328_supplementary_data.docx]

**Supplemental Figure 1.** Piperacillin-Tazobactam Activity against *Escherichia coli* (8,750) from US Medical Centers Stratified by Ceftriaxone Susceptibility (2020-2022)^a^

^a^Enterobacterales were considered susceptible if the ceftriaxone MIC value was ≤1 mg/L. Enterobacterales were considered non-susceptible if the ceftriaxone MIC value was ≥2 mg/L [46].

Abbreviations: CRO-S: ceftriaxone susceptible, CRO-NS: ceftriaxone non-susceptible.

**Supplemental Figure 2.** Piperacillin-tazobactam activity against *Klebsiella pneumoniae* (5,436) from US Medical Centers Stratified by Ceftriaxone Susceptibility (2020-2022)^a^

^a^Enterobacterales were considered susceptible if the ceftriaxone MIC value was ≤1 mg/L. Enterobacterales were considered non-susceptible if the ceftriaxone MIC value was ≥2 mg/L [50].

Abbreviations: CRO-S: ceftriaxone susceptible, CRO-NS: ceftriaxone non-susceptible.

**Supplemental Figure 3.** Piperacillin-Tazobactam Activity against *P. mirabilis* (n=2,187) from United States Medical Centers from 2020-2022, Stratified by Ceftriaxone Susceptibility^a^

^a^Enterobacterales were considered susceptible if the ceftriaxone MIC value was ≤1 mg/L. Enterobacterales were considered non-susceptible if the ceftriaxone MIC value was ≥2 mg/L [29].

**Supplemental Table 1.** Tentative TZP Epidemiological Cutoff (ECOFF) Values for *E. coli* , *K. pneumoniae*, *K. oxytoca*, and *P. mirabilis* Isolates Collected from United States Medical Centers from 2020-2022

| **Enterobacterales Pathogen (n)** | **Tentative TZP ECOFF Values at Increasing Thresholds Based on SENTRY Antimicrobial Surveillance Program Data** | | | | |
| --- | --- | --- | --- | --- | --- |
|  | 95.0% | 97.5% | 99.0% | 99.5% | 99.9% |
| *E. coli* (n=8,750) | 4 | 4 | 4 | 8 | 8 |
| *K. pneumoniae* (n=5,436) | 8 | 8 | 8 | 8 | 16 |
| *K. oxytoca* (n=1,597) | 4 | 4 | 4 | 8 | 8 |
| *P. mirabilis* (n=5,436) | 0.5 | 0.5 | 1 | 1 | 1 |

**Supplemental Table 2:** The probabilities of intermittent- and extended-infusion TZP dosing regimens in achieving tazobactam exposures associated with stasis and 1 log_10_ killing using critically-ill [48] PK data in subjects with estimated creatinine clearances of 60-100 mL/min [27]

**A**: **Free tazobactam concentrations above the TZP MIC value for 64% of dosing interval (stasis)**

| **Tazobactam 500 mg every six hours using Critically Ill patients’ pharmacokinetic data** | | | | | | | | | | | | | | | |
| --- | --- | --- | --- | --- | --- | --- | --- | --- | --- | --- | --- | --- | --- | --- | --- |
|  | 30-minute infusion at TZP MIC (mg/L) | | | | | | | 3-hour infusion at TZP MIC (mg/L) | | | | | | | |
| **CL_CR_**  **(mL/min)** | 0.25 | 0.5 | 1 | 2 | 4 | 8 | 16 | 0.25 | 0.5 | 1 | 2 | 4 | 8 | 16 |  |
| **60** | 100 | 99 | 98 | 94 | 83 | 52 | 0 | 100 | 100 | 100 | 99 | 97 | 79 | <1 |  |
| **80** | 99 | 99 | 97 | 92 | 81 | 46 | 0 | 100 | 100 | 100 | 99 | 95 | 74 | <1 |  |
| **100** | 99 | 98 | 96 | 90 | 77 | 42 | 0 | 100 | 100 | 100 | 99 | 94 | 69 | <1 |  |

**B: Free tazobactam concentrations above the TZP MIC value for 77% of dosing interval (1 log_10_ CFU reduction target)**

| **Tazobactam 500 mg every six hours using critically ill patients’ pharmacokinetic data** | | | | | | | | | | | | | | |
| --- | --- | --- | --- | --- | --- | --- | --- | --- | --- | --- | --- | --- | --- | --- |
|  | 30-minute infusion at TZP MIC (mg/L) | | | | | | | 3-hour infusion at TZP MIC (mg/L) | | | | | | |
| **CL_CR_**  **(mL/min)** | 0.25 | 0.5 | 1 | 2 | 4 | 8 | 16 | 0.25 | 0.5 | 1 | 2 | 4 | 8 | 16 |
| **60** | 99 | 98 | 96 | 91 | 77 | 41 | <1 | 100 | 100 | 99 | 97 | 90 | 62 | <1 |
| **80** | 98 | 97 | 94 | 87 | 71 | 35 | 0 | 100 | 100 | 99 | 96 | 88 | 55 | <1 |
| **100** | 98 | 96 | 93 | 85 | 68 | 31 | 0 | 100 | 99 | 98 | 95 | 85 | 52 | <1 |

**Abbreviations:** *f*T > MIC_TZP_, the percentage of time during the dosing interval that free-drug tazobactam concentrations exceeded the TZP MIC; TZP, piperacillin/tazobactam; MIC, minimum inhibitory concentration; CL_CR_, creatinine clearance.

**Supplemental Table 3.** Probability of PK/PD Target Attainment of Piperacillin % fT>MIC of 50% of Standard vs Extended-infusion Piperacillin/Tazobactam by Creatinine Clearance and Piperacillin/Tazobactam MIC Using Previously Published Population Pharmacokinetic Model [59]

| **TZP Regimen** | **CL_CR_ (ml/min)** | **TZP MIC Value (mg/L)** | | | | | | |
| --- | --- | --- | --- | --- | --- | --- | --- | --- |
|  |  | **0.25** | **0.5** | **1** | **2** | **4** | **8** | **16** |
| **4.5 IV (0.5 h infusion) every 6 h** | **120** | 0.96 | 0.94 | 0.90 | 0.83 | 0.73 | 0.57 | 0.36 |
|  | **100** | 0.98 | 0.96 | 0.93 | 0.88 | 0.81 | 0.67 | 0.46 |
|  | **80** | 0.99 | 0.98 | 0.96 | 0.93 | 0.87 | 0.77 | 0.58 |
|  | **60** | 0.99 | 0.99 | 0.98 | 0.96 | 0.92 | 0.84 | 0.70 |
|  | **40** | 0.99 | 0.99 | 0.99 | 0.98 | 0.96 | 0.92 | 0.84 |
|  | **20** | 0.99 | 0.99 | 0.99 | 0.99 | 0.98 | 0.96 | 0.91 |
| **4.5 IV (4-h infusion) every 8 h** | **120** | 0.99 | 0.99 | 0.99 | 0.99 | 0.99 | 0.98 | 0.82 |
|  | **100** | 0.99 | 0.99 | 0.99 | 0.99 | 0.99 | 0.99 | 0.89 |
|  | **80** | 0.99 | 0.99 | 0.99 | 0.99 | 0.99 | 0.99 | 0.93 |
|  | **60** | 0.99 | 0.99 | 0.99 | 0.99 | 0.99 | 0.99 | 0.96 |
|  | **40** | 0.99 | 0.99 | 0.99 | 0.99 | 0.99 | 0.99 | 0.99 |
|  | **20** | 0.99 | 0.99 | 0.99 | 0.99 | 0.99 | 0.99 | 0.99 |
| **4.5 IV (3-h infusion) every 6 h** | **120** | 0.99 | 0.99 | 0.99 | 0.99 | 0.99 | 0.99 | 0.93 |
|  | **100** | 0.99 | 0.99 | 0.99 | 0.99 | 0.99 | 0.99 | 0.96 |
|  | **80** | 0.99 | 0.99 | 0.99 | 0.99 | 0.99 | 0.99 | 0.98 |
|  | **60** | 0.99 | 0.99 | 0.99 | 0.99 | 0.99 | 0.99 | 0.99 |
|  | **40** | 0.99 | 0.99 | 0.99 | 0.99 | 0.99 | 0.99 | 0.99 |
|  | **20** | 0.99 | 0.99 | 0.99 | 0.99 | 0.99 | 0.99 | 0.99 |

**Abbreviations**: 50% fT>MIC, 50% time that piperacillin free-drug plasma concentrations exceed the minimum inhibitory value during the dosing interval; TZP, piperacillin/tazobactam; CL_CR_, creatinine clearance; MIC, minimum inhibitory value IV, intravenous; h, hours.
